# Effect of Nonselective His Bundle Pacing on Delayed Myocardial Activation in Left-axis Deviation and Left Bundle Branch Block

**DOI:** 10.19102/icrm.2021.120702

**Published:** 2021-07-15

**Authors:** Rehan Mahmud, Shakeel Jamal, Stacey Kukla, Brenda Harris

**Affiliations:** ^1^McLaren Bay Region Medical Center, Bay City, MI, USA

**Keywords:** Nonselective His bundle pacing, left-axis deviation, left bundle branch block

## Abstract

It has been suggested that nonselective His bundle pacing (NS-HBP) corrects terminal conduction delay in right bundle branch block by early excitation of the right ventricular free wall. A similar analysis of NS-HBP, in patients with left bundle branch block (LBBB) and left-axis deviation (LAD) has not been done. Therefore, we compared the baseline QRS parameters in LAD and LBBB during NS-HBP and selective HBP (S-HBP). In LAD patients (n = 16), NS-HBP normalized the QRS axis from −35° ± 10° to 30° ± 34° (p < 0.01) and increased the lead 1 voltage (L1V) from 0.55 ± 0.3 mV to 0.88 ± 0.2 mV (p < 0.001) without increasing the peak lateral wall activation time (PLWAT) (p = not significant). In 23 of 41 LBBB patients, NS-HBP decreased the prolonged PLWAT by 73 ms (p < 0.0001), resolved the mid-QRS notch, normalized the QRS axis, and increased the L1V from 0.5 ± 0.3 mV to 1.15 ± 0.3 mV (p < 0.0001). In the remaining 18 LBBB patients, NS-HBP did not resolve the mid-QRS notch; however, the peak septal activation time decreased by 45 ms (p < 0.0001), PLWAT decreased by 53 ms (p < 0.0001), L1V increased from 0.5 ± 0.3 mV to 0.87 ± 0.4 mV (p < 0.0001), and the QRS axis normalized. All patients who developed S-HBP at lower pacing showed uncorrected LBBB (n = 6) or LAD (n = 7). In conclusion, NS-HBP, which causes myocardial activation in advance of simultaneously initiated S-HBP, results in a paced QRS complex with a normal axis and shorter activation times and restores the L1V in patients with LAD and LBBB. In some patients, a mid-QRS notch was seen with NS-HBP, which suggests fusion with S-HBP, which conducts without LBBB correction. A higher L1V in association with a shorter PLWAT and a normal QRS axis suggests that a more organized degree of left ventricular activation occurs with NS-HBP as compared to LBBB.

## Introduction

There is growing awareness that the site of left bundle branch block (LBBB) may be distal to the His bundle pacing (HBP) site.^1^ This realization has led to considerable interest in pacing the left bundle itself.^2–4^ However, HBP continues to show favorable outcomes in LBBB.^5–9^

It was recently reported that nonselective (NS) HBP (NS-HBP) corrected terminal conduction delay in right bundle branch block (RBBB), possibly by activating early the delayed right ventricular free wall depolarization.^10^ It is not known whether NS-HBP may similarly correct areas of delayed myocardial activation in patients with left-axis deviation (LAD)^11^ or LBBB.^12–14^

## Methods

This was a single-arm observational study designed to evaluate the effects of selective HBP (S-HBP) and NS-HBP in patients with LBBB or LAD.

### Definitions

Patients with LBBB were defined as those with a QRS duration of 140 ms or more with a delayed intrinsicoid deflection of 70 ms or more, together with mid-QRS notching in two or more leads (I, II, AVL, V5, or V6).^13^ Patients with LAD were defined as those with a QRS axis between −30° and −90° and a QRS duration of less than 120 ms. S-HBP was defined as a QRS complex nearly identical to the baseline QRS and a stimulus–QRS interval greater than or equal to the H–V interval. Finally, NS-HBP was defined as a stimulus–QRS interval less than the H–V interval with initial slurring or a delta wave that begins following the pacing stimulus in one or more electrocardiogram (ECG) leads.

### Patient selection

Of 300 patients who underwent HBP, 42 patients met the criteria for LBBB as previously described.^13^ One of these 42 patients showed S-HBP at a higher voltage with complete correction of LBBB and was subsequently excluded from the analysis as pacing was clearly distal to the site of the block. The remaining 41 patients showed NS-HBP at a higher voltage and, of these, six patients showed S-HBP at a lower voltage. Two of the 41 patients showed rate-related LBBB; in these patients, HBP was performed at rates greater than that at which LBBB developed.

Separately, among 28 patients with LAD, 12 had a QRS duration of more than 120 ms and were excluded; the remaining 16 patients were analyzed. All showed NS-HBP at higher voltages; of these, six transitioned to S-HBP at a lower voltage.

### Measurements

From LAD patients, the following measurements were collected: (1) His or stimulus to peak lateral wall activation time (PLWAT), (2) QRS axis, (3) lead 1 voltage (L1V), and (4) V lead transition. Separately, among LBBB patients, the following measurements were gathered where possible: (1) from the His or pacing stimulus to the end of the QRS interval (for QRS activation time), (2) from the His or pacing stimulus to the first notch, (3) from the His or pacing stimulus to the second notch or to the peak lead 1 QRS complex (in the absence of a notch), (4) notch duration, (5) the lead I QRS first notch voltage for septal voltage, (6) the lead I QRS second notch voltage [for the left ventricular (LV) lateral wall voltage], (7) frontal QRS axis, and (8) V lead transition. All native and paced measurements were performed after active fixation of the lead.

In patients with LBBB, the first notch was considered the peak septal activation time^12,13^ and the second notch was considered the peak LV lateral wall activation time,^12,13^ respectively. Activation times were measured from the His bundle electrogram (obtained from the pacing site) or from the onset of the pacing stimulus. The H–V intervals were measured in both the LAD and LBBB study groups.

In summary, patients who met the criteria for LBBB or LAD diagnosis underwent HBP starting at 5 V, which was then decreased in 1-V increments. With each decrement, a 12-lead ECG (filter setting: 0–100 Hz) as well as His bundle electrograms were recorded and stored on the CardioLab signal acquisition and recording system (GE Healthcare, Chicago, IL, USA). Study data, including QRS morphology, were analyzed offline on a review monitor; all measurements were made using CardioLab’s multi-leg calipers.

Statistical analysis was performed using an online calculator (GraphPad, San Diego, CA, USA) with a paired t-test.

All patient data were deidentified, and all patients gave consent for the use of their data for publication. Approval for analyzing the stored data was obtained from the appropriate institutional review board.

## Results

**Figure 1:**
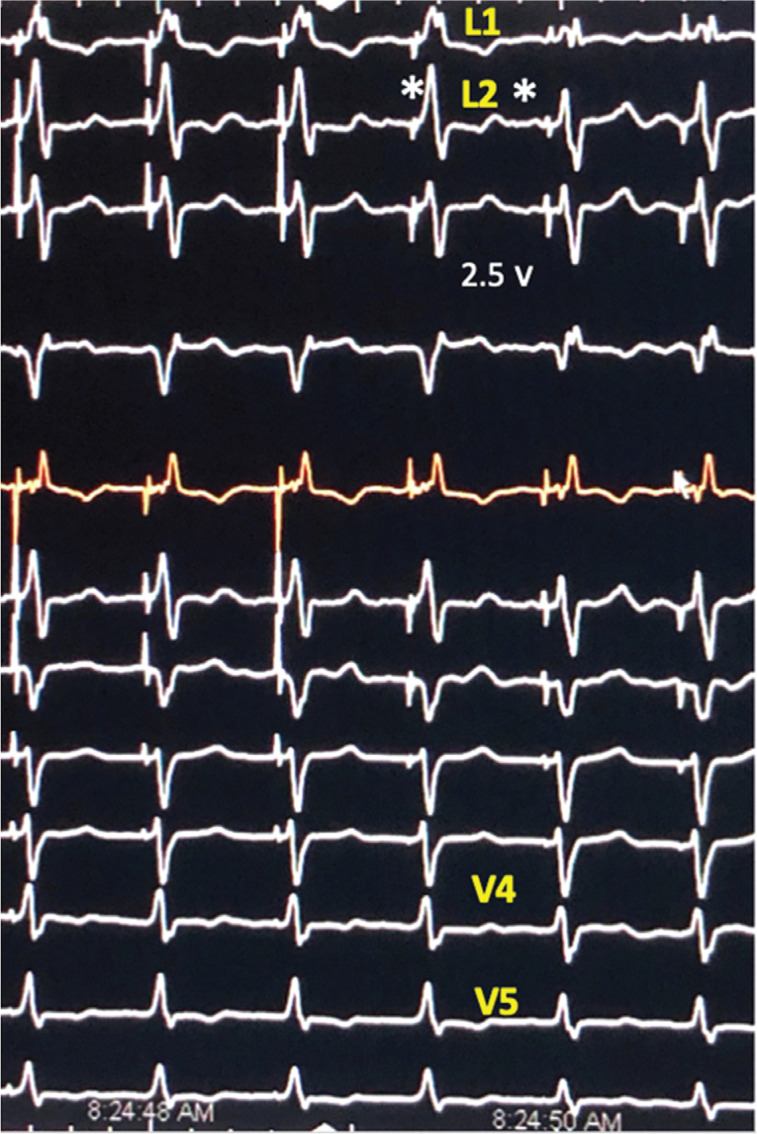
Effect of NS-HBP on isolated LAD. Twelve-lead ECG panel, 25 mm/s. The transition from NS-HBP to S-HBP is shown, marked by an asterisk. Note the fall in L1V with S-HBP. A shift in the QRS axis occurred from 5° to −30° and late transition is seen in V leads.

In patients with LAD (n = 16) (H–V interval: 42 ± 12 ms), NS-HBP produced a significant 58° ± 18° shift in the QRS axis from −35° ± 10° to 30° ± 34° (p < 0.01) together with a shift in chest leads from V6 to V4 **(Table 1 and Figure 1)**. L1V increased from 0.55 ± 0.3 mV to 0.88 ± 0.2 mV (p < 0.001); however, baseline His to PLWAT (102 ± 19 ms) was not significantly different compared to stimulus to PLWAT NS-HBP (92 ± 10 ms) **(Table 1)**. S-HBP was seen at a lower pacing voltage in seven of 16 patients **(Figure 1)**; all showed uncorrected LAD.

**Figure 2:**
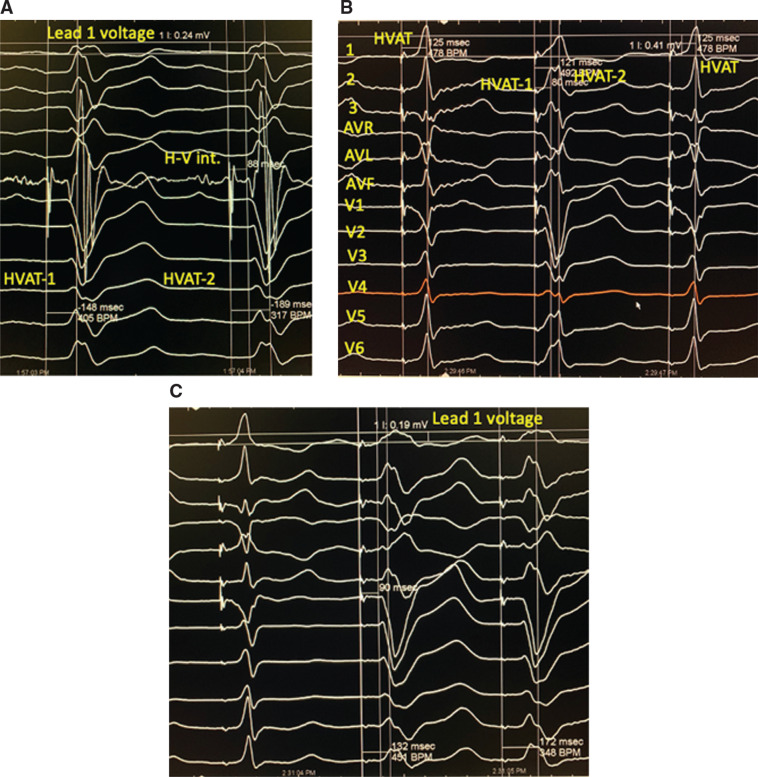
Comparison of baseline LBBB with NS-HBP with and without a notch and S-HBP. Recordings in the figure were made at 100 mm/s. **A:** At baseline LBBB, the H–V interval was 88 ms, the interval from the His bundle electrogram to the septal notch was 148 ms, and the interval from the His bundle electrogram to the lateral wall was 189 ms. The L1V was 0.24 mV. The V lead transition occurred in V6. **B:** NS-HBP complex (first and third beats) without evidence of septal notch. The stimulus to lateral wall interval was reduced by 64 ms to 125 ms, L1V increased to 0.67 V, and the QRS transition is now in V4. As the pacing voltage is reduced, the second beat shows a wide complex with a mid-QRS notch in V6; however, the stimulus to septal notch interval of 80 ms and the stimulus to lateral wall interval of 121 ms were less than those in LBBB. The L1V is higher than in LBBB and less than in NS-HBP, and the QRS axis is normal. **C:** The transition from NS-HBP to S-HBP and complete LBBB are seen as the paced impulse now conducts exclusively via the His bundle and blocks in the left bundle branch. HVAT: His Purkinje system ventricular-activation time.

**Figure 3:**
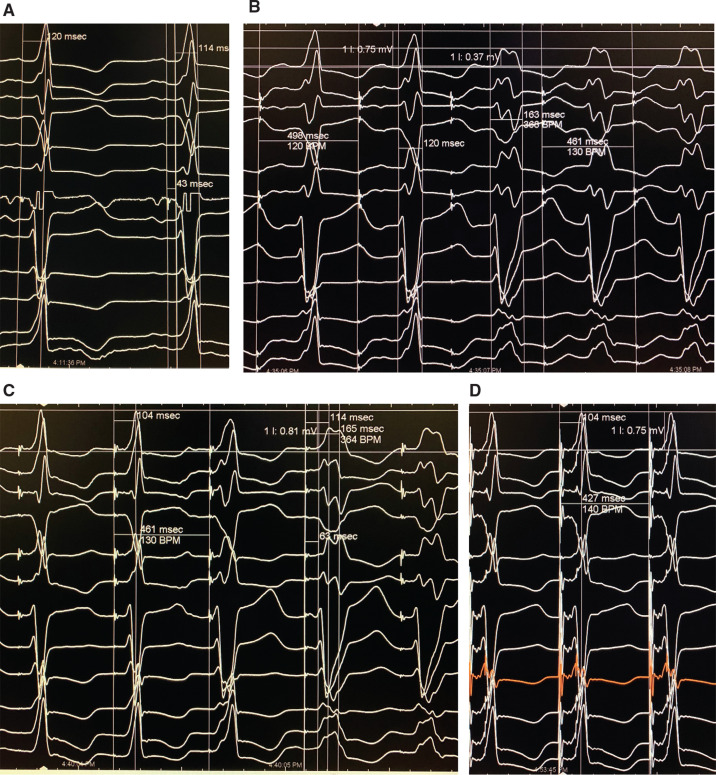
NS-HBP and S-HBP in a patient with rate-related LBBB. Recordings in the figure are presented at 100 mm/s. **A:** A sinus beat with an H–V interval of 43 ms, a His to peak QRS interval of 120 ms, and a QRS interval of 114 ms. **B:** Atrial pacing at 120 bpm resulted in rate-related LBBB. Note the notched wide QRS, decrease in L1V, leftward QRS axis, and loss of R-waves in the chest leads. **C:** HBP at 130 bpm. NS-HBP (first two beats) shows the paced complex to be very similar to the native QRS interval in **A** without LBBB; as the pacing voltage is reduced from 2.0 to 1.8 V, the third beat shows widening of the QRS interval followed by transition from NS-HBP to S-HBP. The “delta” wave is prolonged (third beat) and lost in the next beat as conduction block occurs in the NS-HBP pathway. In the last two beats, S-HBP is seen with uncorrected LBBB, which is identical to the rate-related LBBB in **B**. **D:** Normal NS-HBP at 140 bpm. Note that the delta waves in the NS-HBP complex are subtle and seen in the initial portion of leads 2, 3, and AVF.

In the LBBB patient group (n = 41), the baseline QRS duration was 172 ± 26 ms and the H–V interval was 73 ± 29 ms. All 41 patients showed NS-HBP at 5-V pacing and a loss of pacing occurred at a mean of 1.6 V. S-HBP was seen in six patients at a lower pacing voltage and showed uncorrected LBBB **(Figures 2 and 3)**. However, in the same patient, “correction” of LBBB was observed.

**Figure 4:**
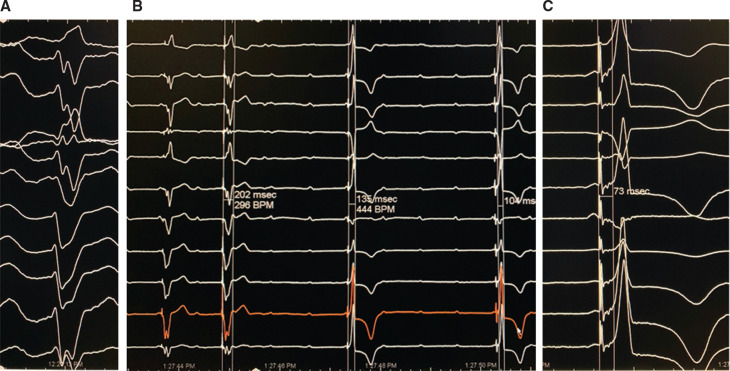
Comparison of LBBB and NS-HBP in iatrogenic complete heart block. **A:** LBBB with LAD (at 100 mm/s). **B:** Complete heart block, which occurred during active fixation of the His bundle lead (at 25 mm/s to allow for more complexes to be seen). RV paced complexes (first two beats) can be compared to NS-HBP complexes (third and fourth beats). **C:** The complex is likely caused exclusively by NS-HBP given the complete heart block. As compared with LBBB, the NS-HBP complex has a narrow QRS, a higher L1 voltage, a normal frontal QRS axis, and R-waves in the anterior chest leads.

**Figure 5:**
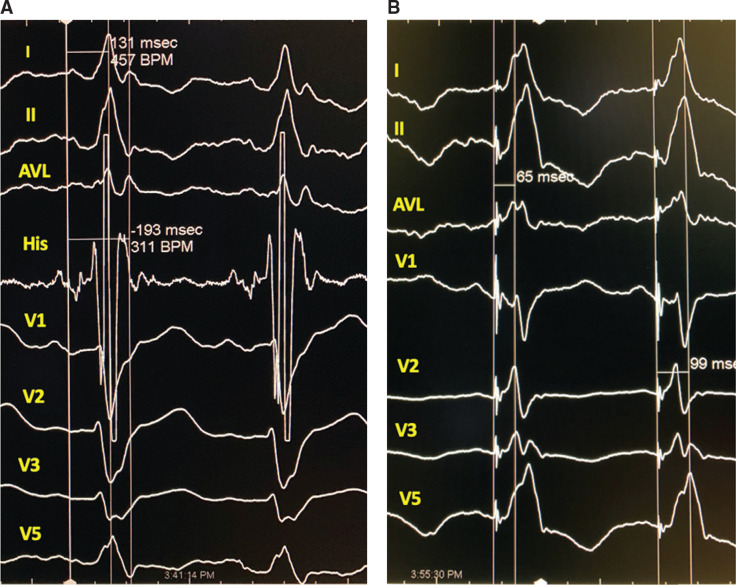
NS-HBP showing fusion with the underlying LBBB. Recordings in the figure are presented at 100 mm/s. **A:** LBBB with a His to septum interval of 131 ms and a His to lateral wall interval of 193 ms. **B:** NS-HBP with mid-QRS notching; however, the stimulus to septum interval was 65 ms and the stimulus to lateral wall interval was 99 ms, which are significantly shorter than in LBBB. Also, L1V is higher and the QRS axis is normal, with a more normal R-wave progression.

With NS-HBP, the QRS complex showed a significant decrease in PLWAT, an increase in L1V, and a normal QRS axis (**Table 2 and Figures 2–5)**. In 23 of 41 patients, NS-HBP showed no mid-QRS notch **(Figures 2–4)**. In the remaining 18 patients, NS-HBP continued to manifest a mid-QRS notch **(Figure 5)**; however, the stimulus to peak LV lateral wall activation time was not significantly different between the two patient groups **(Table 2)**, although patients with NS-HBP without a mid-QRS notch showed a significantly higher L1V and an earlier chest lead transition relative to those with NS-HBP and a mid-QRS notch.

The presence of a notch during NS-HBP allowed a comparison between the His to peak septal-activation time (PSAT) and the stimulus to PSAT, which was significantly shorter by 45 ms with NS-HBP relative to at baseline (p < 0.0001). The septal voltage (of the first notch) measured during NS-HBP was significantly higher than the baseline septal voltage (p < 0.01) **(Table 2)**.

Two of 41 patients showed rate-related LBBB; in these patients, HBP was performed at rates greater than that at which LBBB developed **(Figure 3)**. Also, in these patients, the NS-HBP complex showed QRS with an activation time, QRS axis, and L1V nearly identical to those of the baseline QRS without LBBB. Transition to S-HBP led to an uncorrected rate-related LBBB pattern **(Figure 3)**.

## Discussion

**Figure 6:**
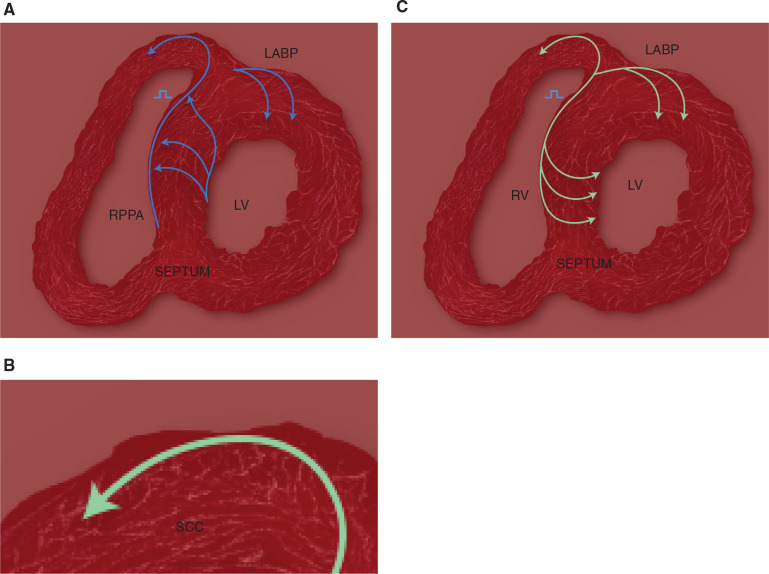
Comparison of excitation pathways in normal activation and in NS-HBP. **A:** A transverse section of the heart with blue arrows representing the normal excitation of the heart.^15^ The left anterior basal paraseptal (LABP) segment, the distal third of the left septum, and the right posterior paraseptal area (RPPA) are the first to be excited.^15^ Also depicted is the normal left to right septal activation. In LBBB, slow septal activation would occur from the right to the left, starting from RPPA, which is the exit point of the right bundle branch.^12–14^ LAD occurs due to delayed activation of the LABP segment. **B:** The recently described specialized conducting cardiomyocytes (SCCs) (shown as pale striations), which make up the distal conduction network.^19–21^ **C:** A likely activation sequence in NS-HBP. The LAD is corrected by early excitation of the LABP segment, while a higher L1V may be explained by the right to left septal activation; however, shorter QRS activation times would require utilization of the specialized conduction network. LV: left ventricle; RV: right ventricle.

Delayed activation of the left anterior paraseptal basal segment results in isolated LAD.^11,12^ In these patients, NS-HBP resulted in a normal QRS axis, a significantly higher L1V, and a stimulus to PLWAT similar to the baseline His to PLWAT **(Figure 1 and Table 1)**. The normal QRS axis suggests that the delayed segment in LAD is excited early in NS-HBP. Thus, similarities to normal excitation of the heart are evident, where the left anterior paraseptal basal segment is also the first to depolarize **(Figure 6)**.^15^ There is little evidence that S-HBP plays a role in the correction of LAD. First, as previously reported in patients with acute and chronic RBBB,^10^ S-HBP, when observed in this study, always showed uncorrected LAD **(Figure 1)**. Second, although NS-HBP and S-HBP wavefronts were initiated simultaneously, myocardial depolarization in NS-HBP was initiated immediately following pacing stimulus, while, with S-HBP, myocardial activation starts following a duration that is greater than or equal in length to the H–V interval (42 ± 12 ms in LAD patients).

Compared to native LBBB, in NS-HBP, the his to peak lateral activation time was significantly shorter, lead 1 voltage was significantly higher, and the QRS axis was more normal **(Figures 2–5 and Table 2)**. The mid-QRS notch, caused by the delayed activation of the septum and lateral wall,^12,13^ was not seen during NS-HBP in 23 of 41 LBBB patients **(Figures 2–4)**. In these patients, the NS-HBP complex, apart from the initial slurring or the “delta wave,” was narrow with a normal QRS axis and without a mid-QRS notch.

Rate-related LBBB provides an opportunity to compare NS-HBP with normal QRS before the development of LBBB in the same patient. **Figure 5** shows that onset of LBBB is associated with a fall in lead 1 voltage, leftward axis, and prolonged septal and lateral wall activation times as well as delayed transition in chest leads. These characteristic changes of LBBB occur as a result of slow, disorganized and abnormally directed activation.^16–18^ The abnormal activation of LBBB is seen with S-HBP but not with NS-HBP, even though, in both instances, the QRS activation would initiate with right to left septal muscle depolarization. **Figure 3** shows that, apart from the subtle delta wave, the NS-HBP complex, at pacing rates which cause LBBB both during atrial pacing and S-HBP, is strikingly similar to patients’ normal QRS. This figure suggests that NS-HBP bypasses the left bundle branch; the site of rate-related conduction block; and, following the delta wave, appears to use the same distal conduction system.^16,19–21^ This postulation is not beyond the realm of possibility, as experimental studies have shown that, in LBBB, Purkinje fibers comprising the distal conduction system are activated earlier on than cardiac myocytes, thereby causing the LV lateral wall to be activated sooner than expected.^16–18^ Use of the distal conduction system would need to be invoked to explain the increase in L1V along with the decrease in PLWAT—that is, why more LV mass is being depolarized in a shorter time **(Tables 1 and 2)**.^16,18–21^

Conceptualizing NS-HBP as a unique pathway may also explain the mid-QRS notch in NS-HBP seen in the remaining 18 patients **(Figure 5 and Table 2)**.

In this study, S-HBP, which is simultaneously activated in NS-HBP, did not correct LBBB **(Figures 1–3)**.^10^ Thus, when conduction in the NS-HBP pathway slows down, perhaps as a result of diffuse Purkinje system disease, such would result in fusion complexes with an S-HBP wavefront and would explain the mid-QRS notch, lower L1V, and late transition in chest leads in these patients. Of interest, the stimulus to PLWATs were not significantly different in the setting of NS-HBP with and without a mid-QRS notch. As compared with baseline LBBB, however, the His to PSAT and the His to peak lateral-activation time were significantly shorter, the L1V was significantly higher, and the QRS axis was normal in NS-HBP **(Figure 5 and Table 2)**.

### Clinical implications, limitations, and the need for further research

Interest in transseptal left-bundle pacing is growing in an effort to place the pacing lead beyond the site of conduction block.^2–4^ Our observations suggest that NS-HBP is an alternative method to achieve the same objective, where the site of conduction block is not a concern and the exact site of pacing is determined by locating the His bundle electrogram.

Past authors have accepted subtle differences in His bundle paced complexes and categorized S-HBP as being in the normal range without normal activation or having a stimulus–Q interval shorter than the H–V interval.^22–24^ In our study, these variances were associated with delta waves in one or more ECG leads and thus were classified as NS-HBP **(Figure 3)**. S-HBP, seen following conduction block in the delta wave, resulted in a QRS complex that was identical to the baseline complex in every lead **(Figures 1–3)**. The fact that S-HBP was always associated with uncorrected LBBB or LAD demonstrated that conduction block was distal to the site of pacing,^10^ and “correction” or “recruitment” with a more narrowly defined S-HBP was rarely observed.^10^

Further research is needed to better understand pacing in an area rich in gap-junction proteins^25^; specialized conducting cardiomyocytes^20,21,26^; and ring tissues, which may participate in the formation of accessory pathways.^27,28^

### Summary

Ventricular activation in NS-HBP, occurring in advance of S-HBP, bypasses the conduction block in bundle branches and results in a unique QRS with a normal activation pattern. Slow conduction in NS-HBP allows for fusion with S-HBP, which continues to manifest an underlying conduction defect.

## Figures and Tables

**Table 1:** Effect of NS-HBP in Patients with LAD

Measurements (n = 16)	Baseline	NS-HBP
His or stimulus to peak lateral wall activation time, ms	102 ± 19	92 ± 10 p = n.s.
QRS axis	−35° ± 10°	+30° ± 34° p < 0.01
Lead 1 voltage, mV	0.55 ± 0.3	0.88 ± 0.2 p < 0.01
V lead transition	V6 ± 1	V4 ± 1 p < 0.01

NS-HBP: nonselective His bundle pacing; n.s.: not significant.

**Table 2:** Comparison of Native LBBB and NS-HBP

	LBBB Native Conduction (n = 41)	NS-HBP Without a Notched Complex (n = 23)	NS-HBP with a Notched Complex (n = 18)
His or stimulus to the end of QRS activation time*, ms	244 ± 43	177 ± 27 (−76) *p < 0.00001	176 ± 26 (−61) *p < 0.00001 **p = n.s.
His or stimulus to peak septal activation time*, ms	136 ± 24	N/A	91 ± 21 (−45) *p < 0.00001
His or stimulus to peak lateral wall activation time*, ms	188 ± 40	119 ± 20 (−73) *p < 0.00001	131 ± 28 (−53) *p < 0.00001 **p = n.s.
Notch duration, ms	48 ± 22	N/A	44 ± 18 (−6.8) *p = n.s.
Lead I QRS voltage septum, mV	0.5 ± 0.3	N/A	0.8 ± 0.4 (0.18) *p < 0.005
Lead I QRS voltage LV lateral wall, mV	0.5 ± 0.3	1.15 ± 0.3 (0.6) *p > 0.0001	0.87 ± 0.4 (0.37) *p > 0.0001 **p < 0.01
Frontal QRS axis	−14° ± 40°	18° ± 32° (30°) *p < 0.02	20° ± 28° (24°) *p < 0.02 **p = n.s.
V lead transition	6 ± 1	3 ± 1 (−2) **p < 0.001	4 ± 1 (−1) *p < 0.001 **p < 0.05

LBBB: left bundle branch block; LV: left ventricular; N/A: not available; n.s.: not significant; NS-HBP: nonselective His bundle pacing.

*Compared to baseline LBBB.

**Compared to NS-HBP without notch.

## References

[1] Upadhyay GA, Cherian T, Shatz DY (2019). Intracardiac delineation of septal conduction in left bundle-branch block patterns. Circulation.

[2] Huang W, Chen X, Su L, Wu S, Xia X, Vijayaraman P (2019). A beginner’s guide to permanent left bundle branch pacing. Heart Rhythm.

[3] Hou X, Qian Z, Wang Y (2019). Feasibility and cardiac synchrony of permanent left bundle branch pacing through the interventricular septum. Europace.

[4] Ponnusamy SS, Arora V, Namboodiri N, Kumar V, Kapoor A, Vijayaraman P (2020). Left bundle branch pacing: a comprehensive review. J Cardiovasc Electrophysiol.

[5] Upadhyay GA, Vijayaraman P, Nayak HM (2019). On-treatment comparison between corrective His bundle pacing and biventricular pacing for cardiac resynchronization: a secondary analysis of the His-SYNC Pilot Trial. Heart Rhythm.

[6] Huang W, Su L, Wu S (2019). Long-term outcomes of his bundle pacing in patients with heart failure with left bundle branch block. Heart.

[7] Lustgarten DL, Crespo EM, Arkhipova-Jenkins I (2015). His-bundle pacing versus biventricular pacing in cardiac resynchronization therapy patients: a crossover design comparison. Heart Rhythm.

[8] Sharma PS, Dandamudi G, Herweg B (2018). Permanent His-bundle pacing as an alternative to biventricular pacing for cardiac resynchronization therapy: a multicenter experience. Heart Rhythm.

[9] Teng AE, Lustgarten DL, Vijayaraman P (2016). Usefulness of his bundle pacing to achieve electrical resynchronization in patients with complete left bundle branch block and the relation between native QRS axis, duration, and normalization. Am J Cardiol.

[10] Mahmud R, Jamal S (2021). Effect of his bundle pacing on right bundle branch block located distal to site of pacing. J Electrocardiol.

[11] Boineau JP, Moore EN, Patterson DF (1973). Relationship between the ECG, ventricular activation, and the ventricular conduction system in ostium primum ASD. Circulation.

[12] Sohi GS, Flowers NC, Horan LG, Sridharan MR, Johnson JC (1983). Comparison of total body surface map depolarization patterns of left bundle branch block and normal axis with left bundle branch block and left-axis deviation. Circulation.

[13] Strauss DG, Selvester RH, Wagner GS (2011). Defining left bundle branch block in the era of cardiac resynchronization therapy. Am J Cardiol.

[14] Walston A, Boineau JP, Spach MS, Ayers CR (1968). Estes EH Jr. Relationship between ventricular depolarization and QRS in right and left bundle branch block. J Electrocardiol.

[15] Durrer D, van Dam RT, Freud GE, Janse MJ, Meijler FL, Arzbaecher RC (1970). Total excitation of the isolated human heart. Circulation.

[16] Venerose RS, Seidenstein M, Stuckey JH, Hoffman BF (1962). Activation of subendocardial Purkinje fibers and muscle fibers of the left septal surface before and after left bundle branch block. Am Heart J.

[17] Morley GE, Danik SB, Bernstein S (2005). Reduced intercellular coupling leads to paradoxical propagation across the Purkinje-ventricular junction and aberrant myocardial activation. Proc Natl Acad Sci U S A.

[18] Becker RA, Scher AM, Erickson RV (1958). Ventricular excitation in experimental left bundle branch block. Am Heart J.

[19] Tawara S (1906). Das Reizleitungssystem des Säugetierherzens. Eine anatomisch-histologische Studie über das Atrioventrikularbündel und die Purkinjeschen Fäden.

[20] De Almeida MC, Araujo M, Duque M, Vilhena V (2017). Crista supraventricularis purkinje network and its relation to intraseptal purkinje network. Anat Rec (Hoboken).

[21] De Almeida MC, Stephenson RS, Anderson RH, Benvenuti LA, Loukas M, Aiello VD (2020). Human subpulmonary infundibulum has an endocardial network of specialized conducting cardiomyocytes. Heart Rhythm.

[22] Vijayaraman P, Dandamudi G, Zanon F (2018). Permanent his bundle pacing: recommendations from a Multicenter His Bundle Pacing Collaborative Working Group for standardization of definitions, implant measurements, and follow-up. Heart Rhythm.

[23] Narula OS (1977). Longitudinal dissociation in the His bundle. Bundle branch block due to asynchronous conduction within the His bundle in man. Circulation.

[24] El-Sherif N, Amay YLF, Schonfield C (1978). Normalization of bundle branch block patterns by distal his bundle pacing. Clinical and experimental evidence of longitudinal dissociation in the pathologic his bundle. Circulation.

[25] Hucker WJ, McCain ML, Laughner JI, Iaizzo PA, Efimov IR (2008). Connexin 43 expression delineates two discrete pathways in the human atrioventricular junction. Anat Rec (Hoboken).

[26] Hulsmans M, Clauss S, Xiao L (2017). Macrophages facilitate electrical conduction in the heart. Cell.

[27] Yanni J, Boyett MR, Anderson RH, Dobrzynski H (2009). The extent of the specialized atrioventricular ring tissues. Heart Rhythm.

[28] Atkinson AJ, Logantha SJ, Hao G (2013). Functional, anatomical, and molecular investigation of the cardiac conduction system and arrhythmogenic atrioventricular ring tissue in the rat heart. J Am Heart Assoc.

